# Ulinastatin alleviates traumatic brain injury by reducing endothelin-1

**DOI:** 10.1515/tnsci-2021-0001

**Published:** 2021-01-07

**Authors:** Ting Liu, Xing-Zhi Liao, Mai-Tao Zhou

**Affiliations:** Jiangsu Province Key Laboratory of Anesthesiology, Xuzhou Medical University, Xuzhou, China

**Keywords:** ulinastatin, traumatic brain injury, brain edema, astrocyte, endothelin-1

## Abstract

**Background:**

Brain edema is one of the major causes of fatality and disability associated with injury and neurosurgical procedures. The goal of this study was to evaluate the effect of ulinastatin (UTI), a protease inhibitor, on astrocytes in a rat model of traumatic brain injury (TBI).

**Methodology:**

A rat model of TBI was established. Animals were randomly divided into 2 groups – one group was treated with normal saline and the second group was treated with UTI (50,000 U/kg). The brain water content and permeability of the blood–brain barrier were assessed in the two groups along with a sham group (no TBI). Expression of the glial fibrillary acidic protein, endthelin-1 (ET-1), vascular endothelial growth factor (VEGF), and matrix metalloproteinase 9 (MMP-9) were measured by immunohistochemistry and western blot. Effect of UTI on ERK and PI3K/AKT signaling pathways was measured by western blot.

**Results:**

UTI significantly decreased the brain water content and extravasation of the Evans blue dye. This attenuation was associated with decreased activation of the astrocytes and ET-1. UTI treatment decreased ERK and Akt activation and inhibited the expression of pro-inflammatory VEGF and MMP-9.

**Conclusion:**

UTI can alleviate brain edema resulting from TBI by inhibiting astrocyte activation and ET-1 production.

## 1 Introduction

Traumatic brain injury (TBI) can lead to serious debilitating complications and fatality [1]. Brain edema is one of the major secondary reactions in TBI resulting in water accumulation in extracellular or intracellular spaces within the brain, in turn resulting in elevated intracranial pressure. Surgical interventions to cure brain edema have limited benefit, hence necessitating other methods of treating brain edema and TBI.

Cellular edema in the astrocytes and/or vasogenic edema related to disruption of the blood–brain barrier (BBB) comprises brain edema and can ultimately lead to neuronal cell death [2,3]. Disruption of the BBB due to brain edema has been shown to result in the development of neurodegenerative diseases such as traumatic epilepsy and Parkinson’s disease [4,5].

Ulinastatin (UTI) has been increasingly used as a protease inhibitor for organ protection [6]. UTI mainly functions by scavenging oxygen free radicals, inhibiting inflammatory reactions, and immune regulation [7]. However, there are few studies on the role and mechanism of UTI in the central nervous system, especially in TBI. UTI was shown to significantly reduce plasma C reactive protein and alleviate brain tissue damage in patients with severe craniocerebral injury [8]. UTI was shown to exert protective effect against brain injury and edema by decreasing the expression and secretion of the pro-inflammatory cytokines, IL-1β and TNF-α, in brain tissue and serum of rats with TBI. TBI-associated brain edema is connected with increased expression of aquaporin-4 at the site of injury [5]; however, how aquaporin-4 contributes to the pathology is not known. However, UTI has been shown to inhibit the expression of aquaporin 4 (AQP4) and alleviate brain edema [10,11]. These studies also indicate that UTI is BBB-permeable.

Cerebral vasospasm caused by traumatic subarachnoid hemorrhage and facilitated by the vasoconstrictor endothelin-1 (ET-1) results in secondary cerebral ischemic edema after brain injury [12]. Intervention with UTI in craniocerebral injury was shown to plasma levels of ET-1, resulting in improvement of hypoperfusion status, cerebral ischemia and hypoxia, and vascular endothelial function [13]. Astrocytes are the main type of glial cells, accounting for more than 50% of the total volume of brain cells. ET-1 produced by activated astrocytes binds to its cognate receptor expressed on astrocytes and causes neuroinflammation [14], potentiated by increased expression of matrix metalloproteinase-9 (MMP-9) and vascular endothelial growth factor-A (VEGF-A) in astrocytes, ultimately resulting in brain edema [15]. In this study, we investigated whether UTI alleviates the severity of TBI by suppressing the astrocyte activity and limiting the production of ET-1 and inflammatory mediators.

## 2 Methods

### 2.1 Establishment of rat model of TBI

Male Sprague-Dawley rats (250–300 g) were housed in humidity- and temperature-controlled housing facility with a 12 h light/dark cycle and free access to rodent chow and water supply. The TBI model was generated using previously described protocol [9]. Rats were anesthetized with 2% pentobarbital sodium (30 mg/kg). After fixing in the prone position, a midline skin incision was made. A trephine bit was then used to generate a 4 mm craniotomy in the right parietal bone between the lambda and bregma, 2 mm dextrolateral to the sagittal suture. The bumper was placed outside the dura mater, the bumper protruded 3 mm from the spiral cylinder, and the brain was contused and lacerated with a 100 g weight from a height of 30 cm, approximately equivalent to a moderate TBI in human. The craniotomy was closed after the blood was stopped. Animals were only returned to their cage after they were bright, alert, and responsive. Then 2% Evans blue liquid was injected into the right femoral vein at a dose of 25 mg/kg, and rats were sacrificed 24 h later. The sham group went through the same protocol except the injury procedure.

### 2.2 Ethical approval

The research related to animals use has been complied with all the relevant national regulations and institutional policies for the care and use of animals.

### 2.3 UTI injection and experimental grouping

Twenty-eight rats undergoing TBI were randomly divided into 2 groups (*n* = 14 each). The sham group also had another 14 rats. The TBI rats were either injected intraperitoneally with UTI (Tianpu Biochemical Pharmaceutical Co., Ltd, Guangdong, China) at a dose of 50,000 U/kg (TBI/UTI) [15] or normal saline (TBI/vehicle) immediately after induction of TBI. The animals were sacrificed after 24 h and brain tissue was resected.

### 2.4 Determination of water content in brain tissue

After 24 h, the resected brain tissues (*n* = 3 per experimental group) were immediately weighed (wet weight) and then dried in an oven at 105°C for 3 days and weighed again (dry weight). The water content of brain tissue was calculated as (wet weight − dry weight)/wet weight × 100%.

### 2.5 Assessment of permeability of BBB using Evans blue dye

Two percent Evans blue dye (Sigma, LA, USA) was injected into the right femoral vein of rats (*n* = 5 per experiment group) at a dose of 25 mg/kg, after 24 h, and allowed to circulate for 1 h. Phosphate buffered saline (0.1 mol/L) was then perfused transcardially. The resected brain tissues were subsequently homogenized in PBS and centrifuged for 30 min at 15,000*g*. An equal volume of trichloroacetic acid was added to the supernatant and incubated at 48°C overnight, followed by centrifugation at 15,000*g* for 30 min. Extravasated Evans blue dye in the supernatant was quantified by measuring absorbance at 615 nm using a spectrophotometer.

### 2.6 Immunohistochemistry (IHC)

Resected brain tissues (*n* = 3 per experiment group) were fixed by immersion in 4% formaldehyde (Beyotime, Zhejiang, China) for 60 min at 37°C before paraffin embedding. These samples were then sectioned at 5 µm thickness via microtome (Model HM310, Microm Inc., USA), dewaxed with xylene, and cleared with a series of changing ethanol concentrations. Blocking was done by incubation in 5% bovine serum albumin (Thermo Fisher Scientific, Carlsbad, CA, USA) and 0.3% Triton- X-100 (Thermo Fisher Scientific) for 1 hour at room temperature (RT). Antibodies used for IHC were glial fibrillary acidic protein (GFAP) (1:100, Abcam, Cambridge, USA), ET-1 (1:50, Abcam), VEGF-A (1:100, Cell Signaling Technology, Cambridge, MA, USA), or MMP-9 (1:100, Cell Signaling Technology). All incubation with primary antibodies were done in 1 BSA overnight at 4°C. Samples were viewed at 100× magnification by light microscopy (BX61, Olympus Optical GmbH, Hamburg, Germany) and imaged (Color View II; Soft Imaging System, Olympus Optical GmbH, Hamburg, Germany).

### 2.7 Western blot analysis

Tissue specimens (*n* = 3 per experiment group) were snap-frozen in liquid nitrogen and homogenized using a mortar and pestle. Lysis was done in RIPA buffer (Beyotime, Zhejiang, China). Protein quantification was done using the BCA assay kit (Thermo Fisher Scientific, CA, USA). Proteins were separated on 12% SDS PAGE. Antibodies used to probe the blots were: GFAP (1:1,000; Abcam), ET-1 (1:2,000; Abcam), VEGF (1:1,000; Cell Signaling Technology), MMP-9 (1:1,000; Cell Signaling Technology), P-ERK1/2 (1:1,000; Cell Signaling Technology), ERK1/2 (1:1,000; Cell Signaling Technology), P-Akt (S473) (1:1,000; Cell Signaling Technology), P-Akt (T308) (1:1,000; Cell Signaling Technology), Akt (1:1,000; Cell Signaling Technology), and β-Actin (1:6,000; Abcam). The β-actin protein was used as a loading control for data normalization. Densitometry analysis was done using the NIH Image J software.

### 2.8 Statistical analysis

Data were presented as mean ± standard error (SD). Data were analyzed using one-way analysis of variance (ANOVA) followed by the Tukey *post hoc* test. *P* < 0.05 was considered as significant.

## 3 Results

### 3.1 UTI alleviates brain edema and prevents disruption of BBB in rat model of TBI

**Figure 1:**
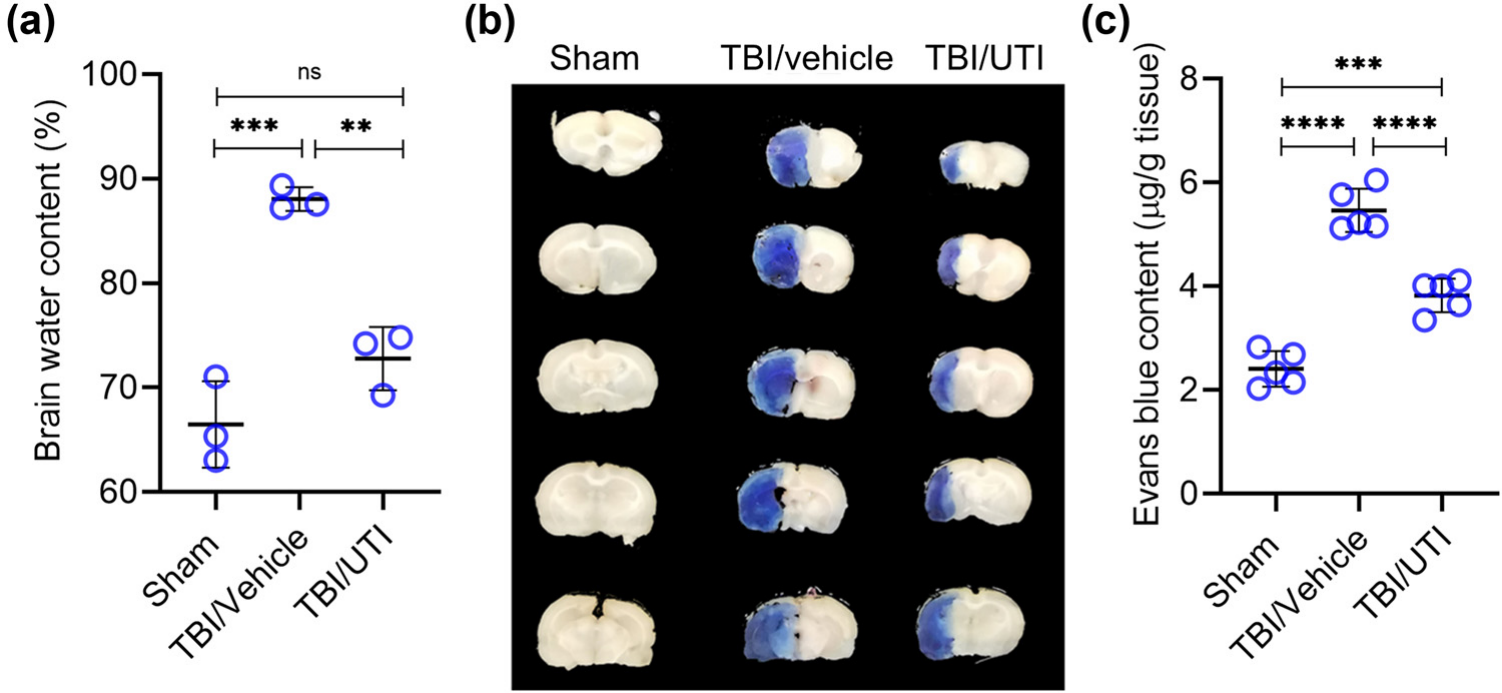
Administration of UTI immediately after induction of TBI alleviates brain edema and prevents BBB disruption. TBI-induced rats were randomly divided into two groups – vehicle control (TBI/vehicle) and UTI (TBI/UTI). The latter group was intraperitoneally injected with 50,000 U/kg of UTI. No TBI was induced in the third sham control group. (a) Brain water content was determined in resected brain tissues after 24 h; (b) the integrity of the BBB was evaluated by Evans blue dye staining; (c) quantification of Evans blue dye extravasation in the different experimental groups. Error bars in (a) and (c) are standard deviation; **p < 0.01, ***p < 0.001, ****p < 0.0001, ns, not significant – one-way ANOVA with Tukey’s *post hoc* test (*n* = 3 in (a) and *n* = 5 in (c)).

Brain water content was significantly increased in rats subjected to UTI compared to the sham control (Figure 1a). Intraperitoneal injection of UTI, administrated immediately after TBI induction, significantly attenuated the increase in brain water content to levels observed in the sham control (Figure 1a). We next evaluated BBB disruption after 24 h using Evans blue dye. Vehicle-treated rats had significantly increased Evans blue dye extravasation in right hemisphere compared with sham rats (Figure 1b and c). In contrast, TBI rats treated with UTI had significantly reduced Evans blue dye extravasation compared with the vehicle-treated TBI rats (Figure 1b and c). However, Evans blue dye extravasation was still significantly higher in the UTI-treated TBI rats compared with the sham control (Figure 1b and c).

### 3.2 UTI inhibits the activity of astrocytes by suppressing expression of ET-1 expression in TBI rats

**Figure 2:**
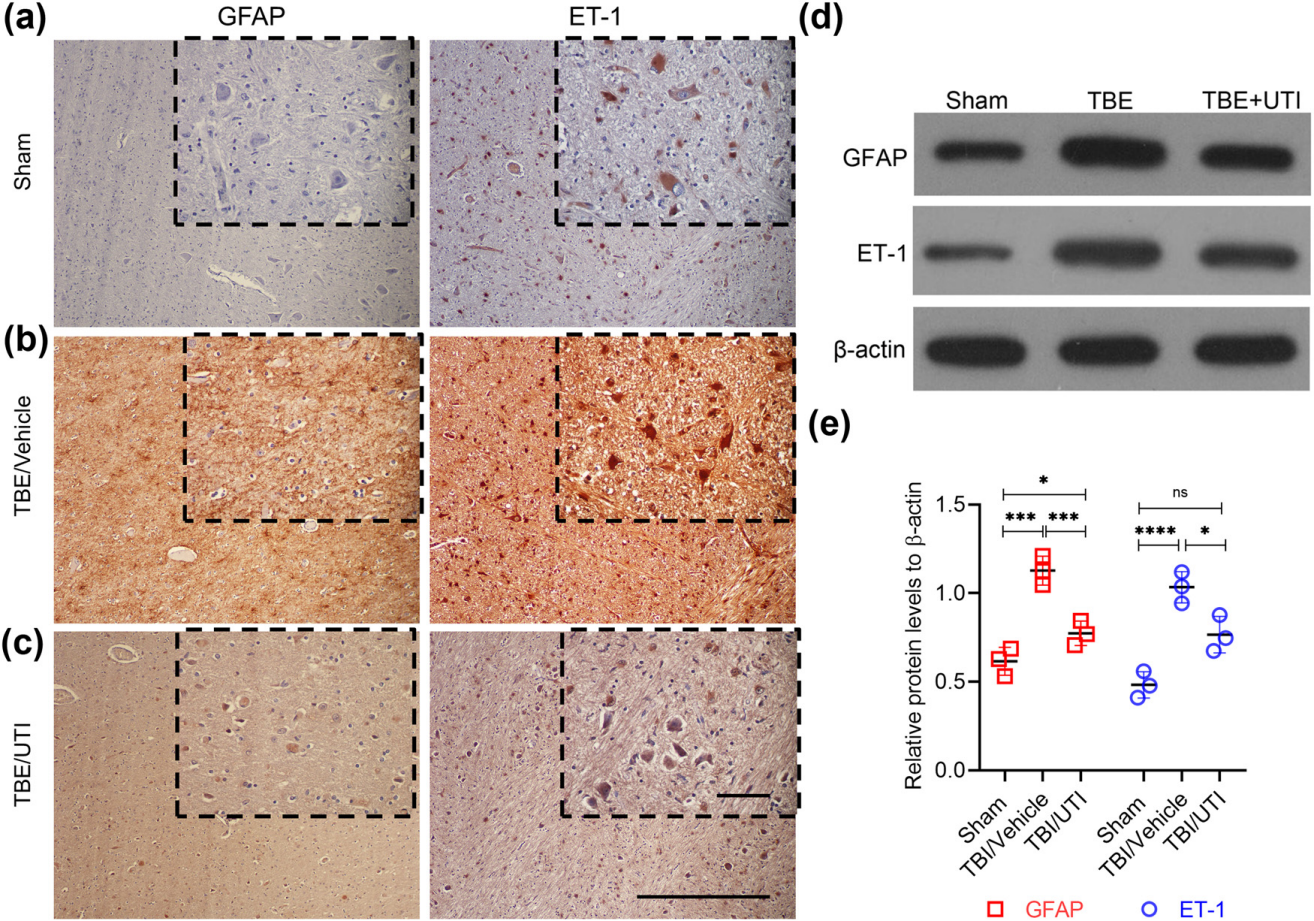
UTI inhibits the activity of astrocytes and decreases expression of ET-1 in the rat brain. (a–d) Representative images of IHC staining of GFAP and ET-1 on resected brain tissues of the different experimental groups (*n* = 3, each group). Insets in (a–d) show images obtained under higher magnification. Scale bar = 30 µm; (d) representative images of immunoblots showing expression of GFAP and ET-1 in the indicated experimental groups. β-Actin was used as a loading control; (e) quantification of relative expression of GFAP and ET-1 in the indicated experimental groups. Densitometry analysis of blots shown in (b) was done using Image J and data were normalized to expression of β-actin. Error bars are standard deviation; *p < 0.05, ***p < 0.001, ****p < 0.0001, ns, not significant – one-way ANOVA with Tukey’s *post hoc* test (*n* = 3).

The GFAP is mainly distributed in the astrocytes. It participates in cell cytoskeleton formation and maintains the tensile strength. GFAP is also used as a marker of astrocyte activation [16]. Activated astrocyte exhibit increased expression of ET-1. To assess the levels of GFAP and ET-1, IHC and western blot analyses were performed. Based on the GFAP level, we found that the activity of astrocytes was significantly higher in TBI/vehicle rats when compared with the sham group (Figure 2a, b, d and e). Similarly, the expression of ET-1 was also significantly high in the TBI/vehicle group compared with the sham group (Figure 2a, b, d and e). However, UTI treatment resulted in significant inhibition of both activation of astrocytes and ET-1 expression (Figure 2c–e). Overall, these results indicate that inhibitory effect of UTI on astrocyte activation in TBI rats is potentially correlated to ET-1 expression.

### 3.3 UTI decreases expression of inflammatory mediators in TBI rats

**Figure 3:**
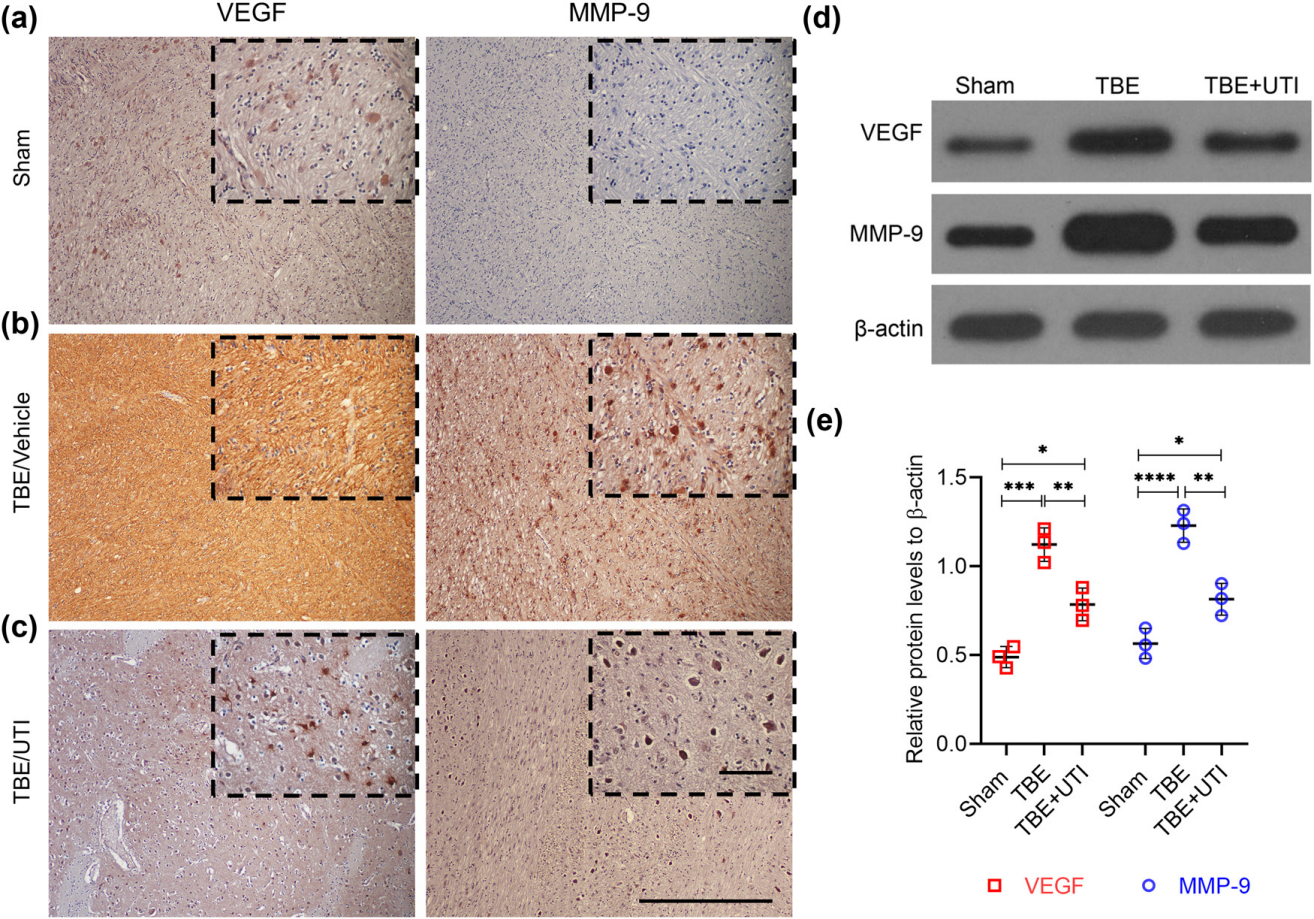
UTI decreases expression of inflammatory mediators in TBI rats. (a–d) Representative images of IHC staining of VEGF and MMP-9 on resected brain tissues of the different experimental groups (*n* = 3, each group). Insets in (a–d) show images obtained under higher magnification. Scale bar = 30 µm; (d) representative images of immunoblots showing expression of VEGF and MMP-9 in the indicated experimental groups. β-Actin was used as a loading control; (e) quantification of relative expression of VEGF and MMP-9 in the indicated experimental groups. Densitometry analysis of blots shown in (d) was done using Image J and data were normalized to expression of β-actin. Error bars are standard deviation; *p < 0.05, **p < 0.01, ***p < 0.001, ****p < 0.0001, ns, not significant – one-way ANOVA with Tukey’s *post hoc* test (*n* = 3).

Inflammatory mediators MMP-9 and VEGF are known to be regulated by ET-1 [15]. Therefore, to verify the fact that the effect of UTI treatment in TBI rats was due to the decreased expression of ET-1, we further assessed the levels of MMP-9 and VEGF. Both IHC and western blot analyses revealed that compared with the sham group, expression of VEGF and MMP-9 was significantly higher in TBI rats (Figure 3a, b, d and e). Administration of UTI resulted in significant decrease in MMP-9 and VEGF (Figure 3c–e). These results provide further evidence that UTI-mediated alleviation of brain edema and BBB disruption in TBI rats is mediated by a decrease in astrocyte activation and ET-1 expression.

### 3.4 UTI inhibits inflammatory mediators in rat astrocytes potentially via inhibiting ERK activation and stimulating the PI3K/AKT pathway

**Figure 4:**
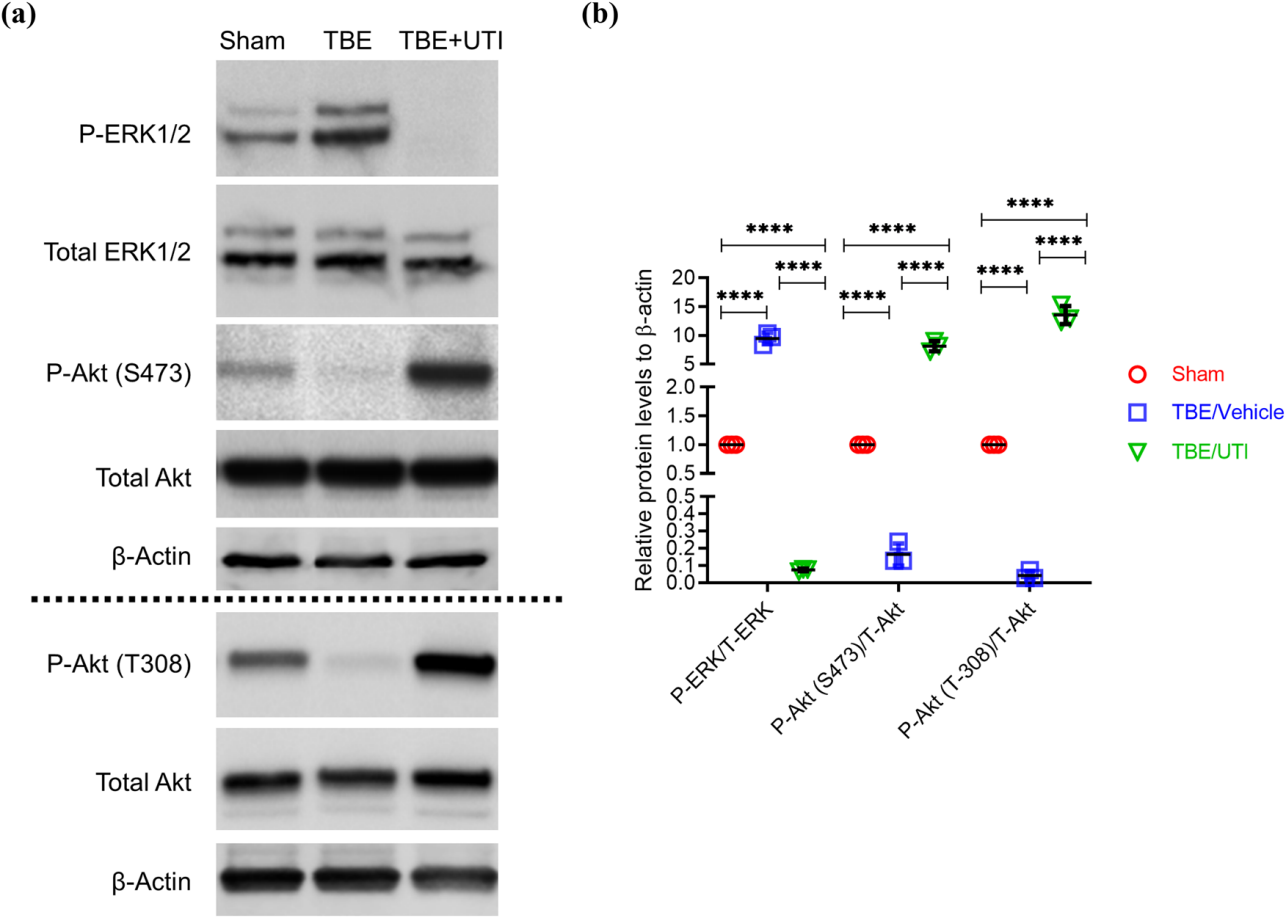
UTI inhibits ERK activation and induces activation of the PI3K/AKT pathway in TBI rats. (a) Representative images of immunoblots showing expression of P-ERK1/2, total ERK1/2, P-Akt (S473), P-Akt (T308), and total Akt in the indicated experimental groups. β-Actin was used as a loading control; (b) quantification of relative expression of P-ERK/total ERK (T-ERK), P-Akt (S473)/total Akt (T-Akt), and P-Akt (T308)/T-Akt in the indicated experimental groups. Densitometry analysis of blots shown in (b) was done using Image J and data were normalized to expression of β-actin. Error bars are standard deviation; ****p < 0.0001 – one-way ANOVA with Tukey’s *post hoc* test (*n* = 3).

Vasoactive peptide ET-1 induces vasoconstriction and proliferation in aortic smooth muscle cells and its receptors function by the activation of ERK and inhibition of the PI3K/Akt pathway [17]. Therefore, we next evaluated if induction of ET-1 in TBI rats is accompanied by the activation of ERK1/2. Results from western blot analysis show that compared with the sham group, TBI rats had significantly higher expression of P-ERK/total ERK (Figure 4a and b). At the same time, using levels of P-AKT (Serine 473)/total AKT and P-AKT (Threonine 308)/total AKT, a significant downregulation of PI3K/AKT pathway was noticed in TBI rats (Figure 4a and b). Administration of UTI resulted in significant decrease in ERK activation along with an increased activation of the PI3K/AKT pathway (Figure 4a and b). Since ERK activation is a read-out of the ET-1 receptor activity, these results provide convincing evidence that UTI alleviates TBI-associated brain edema and BBB disruption in astrocytes via downregulating expression of ET-1 and suppressing the activity of the ET-1 receptor.

## 4 Discussion

Brain edema, increased intracranial pressure, and disruption of BBB are associated to the high incidence of long-term disabilities and mortality rate in cases of TBI [11]. Surgical intervention has limited outcomes, necessitating identification of additional therapeutic options. Results from the current study indicate that administration of UTI might be an attractive regimen to manage TBI.

BBB is mainly composed of microvascular endothelial cells, astrocytes, microglial cells, pericytes, and basement membranes [18]. Incidence of TBI is directly related to the dysfunction of BBB [19]. Disruption of BBB allows intra- and extravascular fluid accumulation, ultimately leading to increased intracranial pressure and vascular edema [20]. AQ4, which is key membrane protein produced by the astrocytes, is critical in maintaining BBB integrity and water balance in the brain. Increased AQ4 results in astrocyte swelling and contributes to the pathogenesis of TBI [21,22,23]. In acute TBI, the absence of AQP4 could alleviate cell edema of astrocytes by reducing the size of lesion [24]. Additionally, matrix metalloproteinases (MMPs) produced by the astrocytes function in disrupting the tight junction of BBB and the basement membrane, thereby further aggravating the cerebral edema [25]. Recently, BBB injury has also been related to neuroinflammation [26]. In hypoxic brain tissue, vascular endothelial cell growth inhibitory factor was shown to reduce inflammation and stabilize BBB by inhibiting the TLR4/NF-κB signaling pathway [27]. Indeed, UTI administration has been shown to decrease expression of AQ4, thus restoring integrity of BBB [9].

Therefore, to study the role and mechanism of UTI in TBI, we primarily focused on its effect on BBB function. Animal experiments and morphological studies confirmed that UTI indeed protected the integrity of BBB and alleviated brain edema. UTI decreased ET-1 expression in astrocytes. ET-1 and nitric oxide (NO) function in maintaining the hemodynamic stability [28]. The bioactivity of ET-1, a member of the ET family, is mediated by vasoconstriction and inflammation [29]. Recently, it was suggested that ET-1 plays a crucial role in both normal development and neurological diseases [30]. In response to cerebral hypoxia or ischemic injury, both ECs and astrocytes activate the release of ET-1 [31]. The secreted ET-1 subsequently induces the secretion of IL-1β in astrocytes, which facilitates the disruption of BBB, cumulatively culminating in cerebral inflammation [32].

TBI stimulates ET-1 release in astrocytes via G-protein coupled ET receptors [33]. The astrocytes are the major cell type that provides functional and structural support to neurons. An *in vitro* study showed that ET-1 exposure led to the overexpression of nitric oxide synthase (iNOS), production of NO, and MMP-9 in astrocytes [34]. The interaction of ET-1 and its receptor activates PI3K/Akt pathway and its downstream partner, nuclear factor-kappa B (NF-κB) [35]. Furthermore, the vascular endothelial growth factor receptor (VEGF-R) is overexpressed in traumatic brain tissues [36]. ET-1 exposure was shown to induce upregulation of VEGF-R, in turn activating VEGF-R1-mediated ERK1/2 signaling in the brain tissues [37]. Results from this study show that administration of UTI inhibited ET-1 expression, MMP-9 and VEGF production, and inactivation of ERK1/2 signaling, while stimulating PI3K/Akt signaling. Hence, UTI is capable of disrupting the different nodes of the cascade involved in the pathogenesis of BBB disruption and brain edema following TBI, making it a potential candidate for further preclinical evaluation in different models of TBI.
