# Erratum to “Ulinastatin alleviates traumatic brain injury by reducing endothelin-1”

**DOI:** 10.1515/tnsci-2020-0205

**Published:** 2021-12-09

**Authors:** Ting Liu, Xing-Zhi Liao, Mai-Tao Zhou

**Affiliations:** Jiangsu Province Key Laboratory of Anesthesiology, Xuzhou Medical University, Xuzhou 22100, China; The 904th Hospital of the Joint Logistics Support Force of PLA, Wuxi, Jiangsu 214044, China

In the published article “Liu T, Liao XZ, Zhou MT. Ulinastatin alleviates traumatic brain injury by reducing endothelin-1. Transl Neurosci. 2021 Jan 7;12(1):1–8. doi: 10.1515/tnsci-2021-0001,” the authors recently found errors in author information and the correct author information is as follows:

Mai-Tao Zhou: Jiangsu Province Key Laboratory of Anesthesiology, Xuzhou Medical University, Xuzhou 221000, China.

Ting Liu: Jiangsu Province Key Laboratory of Anesthesiology, Xuzhou Medical University, Xuzhou 221000, China.

Xing-Zhi Liao: The 904th Hospital of the Joint Logistics Support Force of PLA, Wuxi, Jiangsu 214044, China.

The authors would like to apologize for any inconvenience caused.

